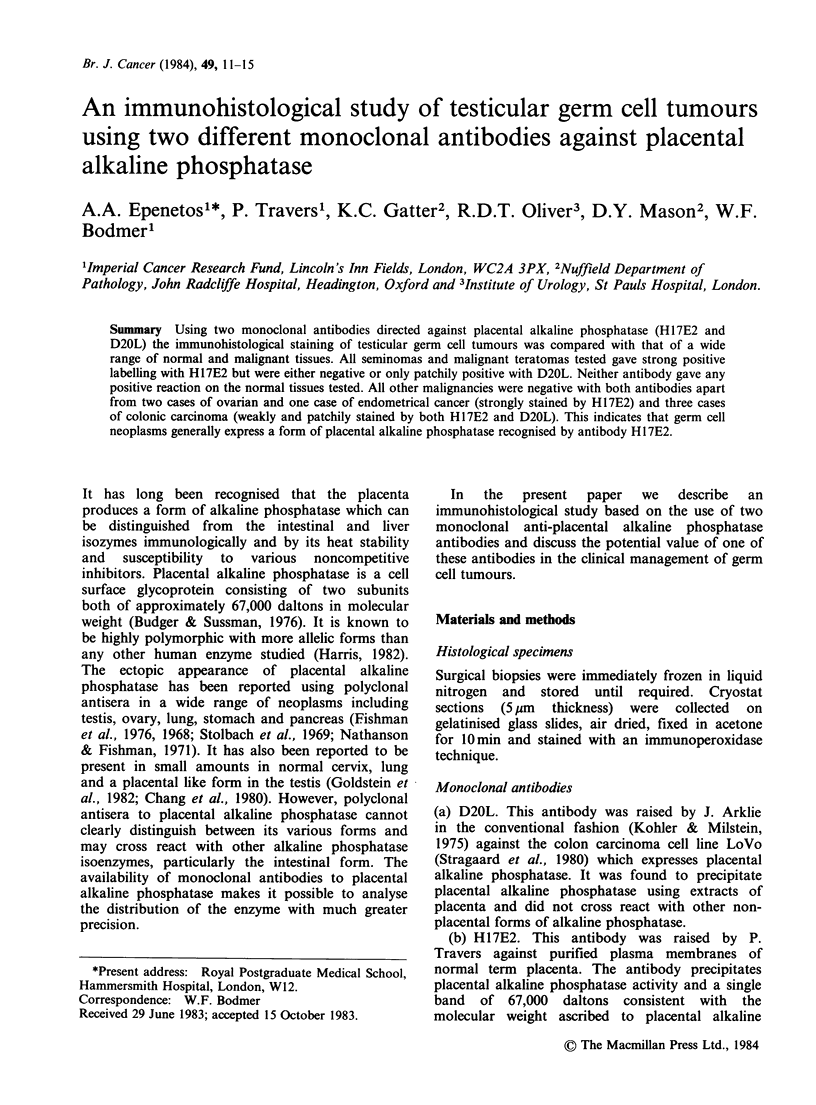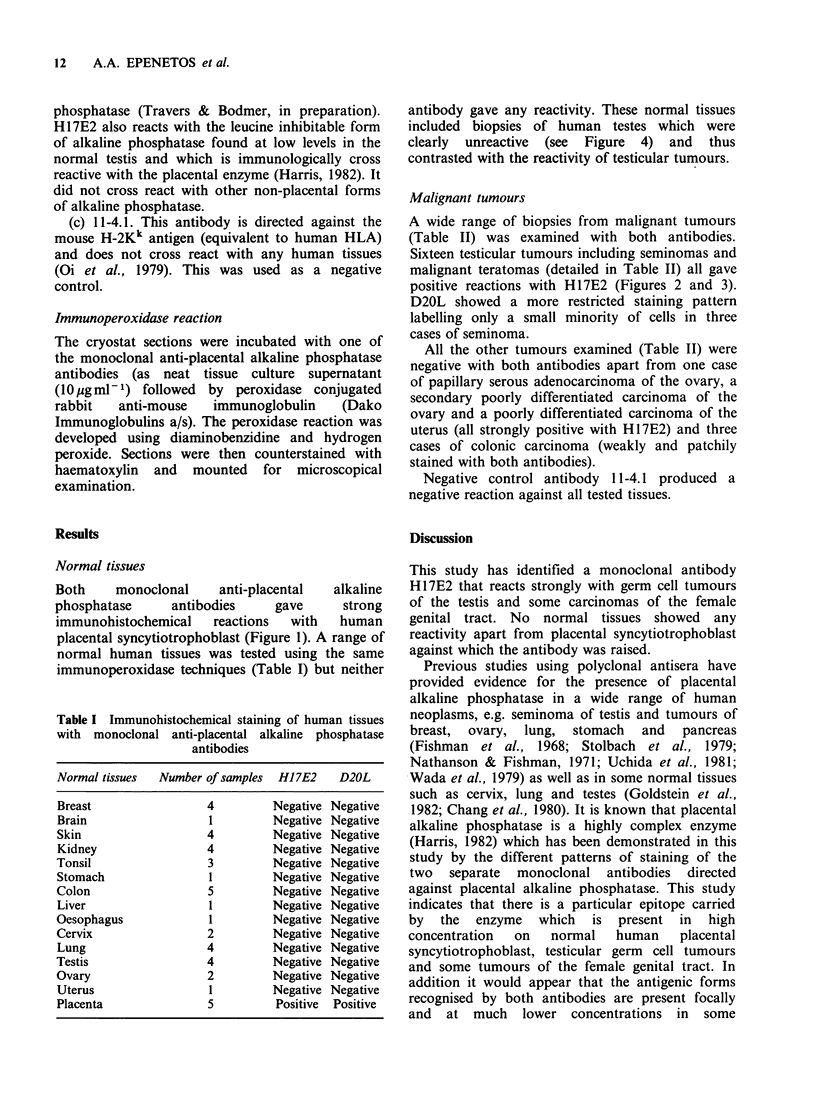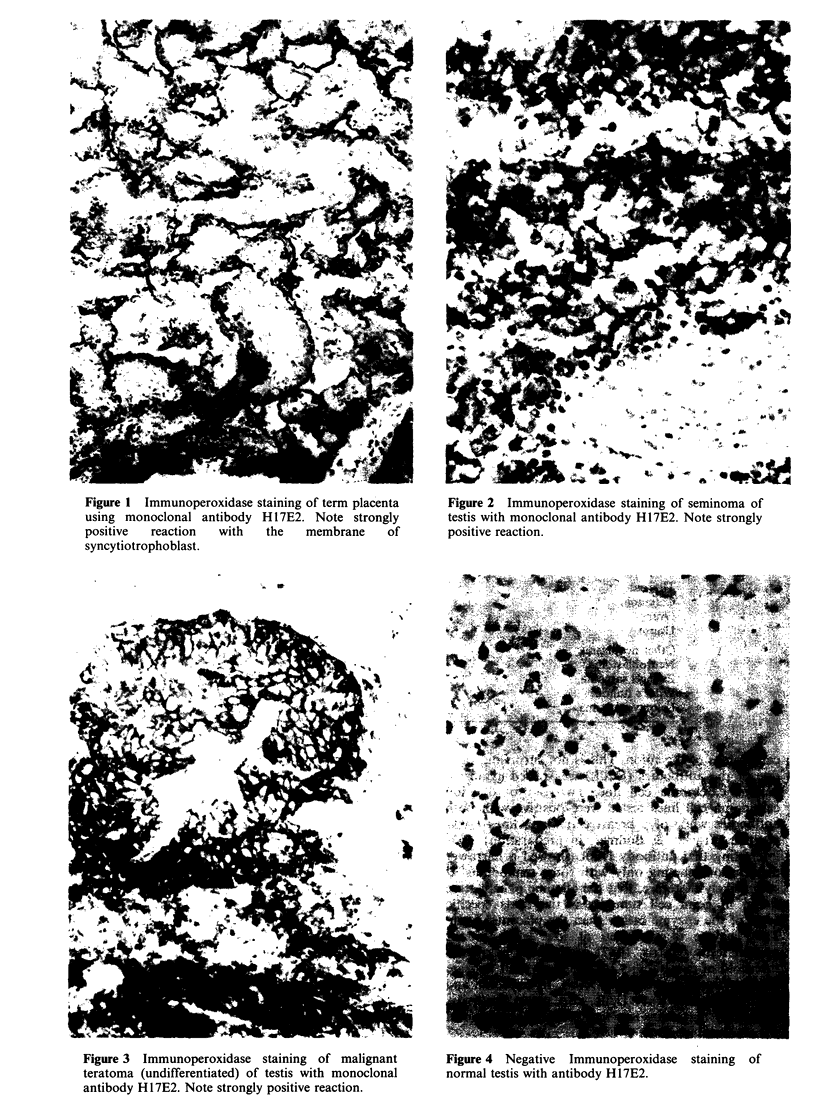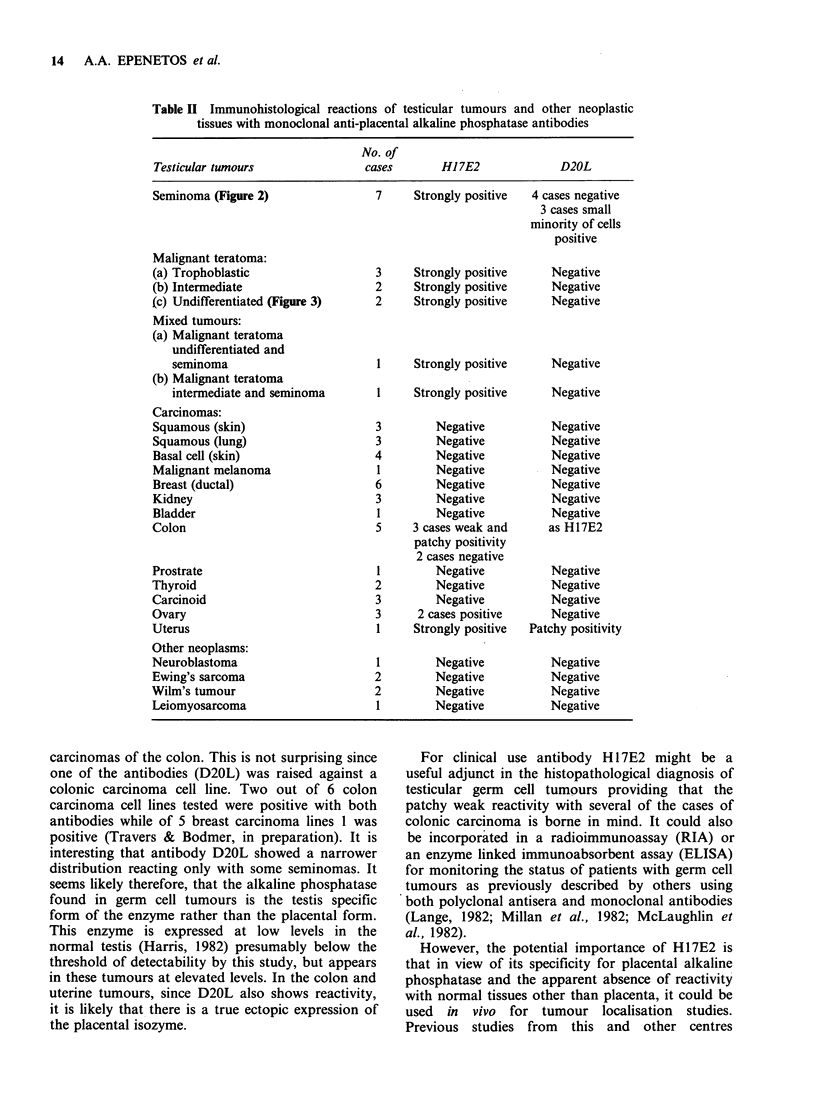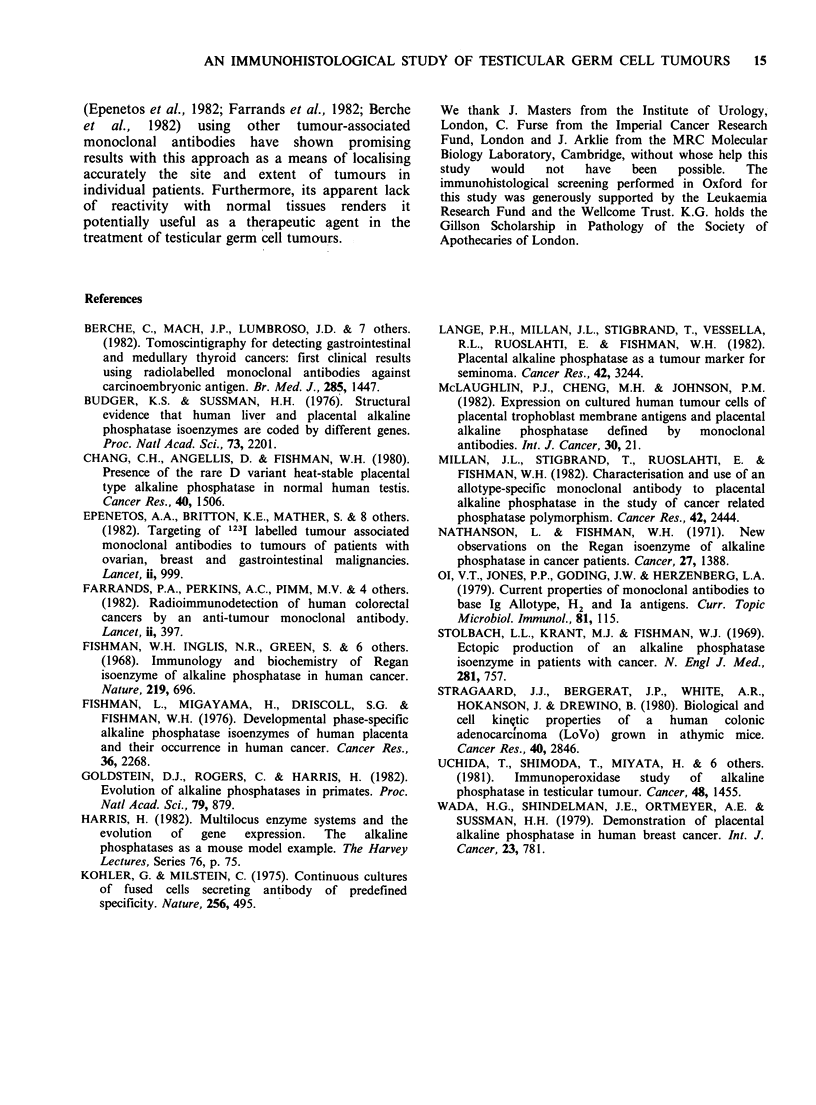# An immunohistological study of testicular germ cell tumours using two different monoclonal antibodies against placental alkaline phosphatase.

**DOI:** 10.1038/bjc.1984.3

**Published:** 1984-01

**Authors:** A. A. Epenetos, P. Travers, K. C. Gatter, R. D. Oliver, D. Y. Mason, W. F. Bodmer

## Abstract

**Images:**


*Br. J. Cancer* (1984), **49**, 11–15

### An immunohistological study of testicular germ cell tumours using two different monoclonal antibodies against placental alkaline phosphatase

A.A. Epenetos[1*], P. Travers[1], K.C. Gatter[2], R.D.T. Oliver[3], D.Y. Mason[2], W.F. Bodmer[1]

[1]*Imperial Cancer Research Fund, Lincoln's Inn Fields, London, WC2A 3PX,* [2]*Nuffield Department of Pathology, John Radcliffe Hospital, Headington, Oxford and* [3]*Institute of Urology, St Pauls Hospital, London.*

**Summary** Using two monoclonal antibodies directed against placental alkaline phosphatase (H17E2 and D20L) the immunohistological staining of testicular germ cell tumours was compared with that of a wide range of normal and malignant tissues. All seminomas and malignant teratomas tested gave strong positive labelling with H17E2 but were either negative or only patchily positive with D20L. Neither antibody gave any positive reaction on the normal tissues tested. All other malignancies were negative with both antibodies apart from two cases of ovarian and one case of endometrical cancer (strongly stained by H17E2) and three cases of colonic carcinoma (weakly and patchily stained by both H17E2 and D20L). This indicates that germ cell neoplasms generally express a form of placental alkaline phosphatase recognised by antibody H17E2.

It has long been recognised that the placenta produces a form of alkaline phosphatase which can be distinguished from the intestinal and liver isozymes immunologically and by its heat stability and susceptibility to various noncompetitive inhibitors. Placental alkaline phosphatase is a cell surface glycoprotein consisting of two subunits both of approximately 67,000 daltons in molecular weight (Budger & Sussman, 1976). It is known to be highly polymorphic with more allelic forms than any other human enzyme studied (Harris, 1982). The ectopic appearance of placental alkaline phosphatase has been reported using polyclonal antisera in a wide range of neoplasms including testis, ovary, lung, stomach and pancreas (Fishman *et al.*, 1976, 1968; Stolbach *et al.*, 1969; Nathanson & Fishman, 1971). It has also been reported to be present in small amounts in normal cervix, lung and a placental like form in the testis (Goldstein *et al.*, 1982; Chang *et al.*, 1980). However, polyclonal antisera to placental alkaline phosphatase cannot clearly distinguish between its various forms and may cross react with other alkaline phosphatase isoenzymes, particularly the intestinal form. The availability of monoclonal antibodies to placental alkaline phosphatase makes it possible to analyse the distribution of the enzyme with much greater precision.

*Present address: Royal Postgraduate Medical School, Hammersmith Hospital, London, W12.
Correspondence: W.F. Bodmer
Received 29 June 1983; accepted 15 October 1983.

In the present paper we describe an immunohistological study based on the use of two monoclonal anti-placental alkaline phosphatase antibodies and discuss the potential value of one of these antibodies in the clinical management of germ cell tumours.

### Materials and methods

### *Histological specimens*

Surgical biopsies were immediately frozen in liquid nitrogen and stored until required. Cryostat sections (5 $\mu$m thickness) were collected on gelatinised glass slides, air dried, fixed in acetone for 10 min and stained with an immunoperoxidase technique.

### *Monoclonal antibodies*

(a) D20L. This antibody was raised by J. Arklie in the conventional fashion (Kohler & Milstein, 1975) against the colon carcinoma cell line LoVo (Stragaard *et al.*, 1980) which expresses placental alkaline phosphatase. It was found to precipitate placental alkaline phosphatase using extracts of placenta and did not cross react with other non-placental forms of alkaline phosphatase.

(b) H17E2. This antibody was raised by P. Travers against purified plasma membranes of normal term placenta. The antibody precipitates placental alkaline phosphatase activity and a single band of 67,000 daltons consistent with the molecular weight ascribed to placental alkaline

© The Macmillan Press Ltd., 1984

phosphatase (Travers & Bodmer, in preparation). H17E2 also reacts with the leucine inhibitable form of alkaline phosphatase found at low levels in the normal testis and which is immunologically cross reactive with the placental enzyme (Harris, 1982). It did not cross react with other non-placental forms of alkaline phosphatase.

(c) 11-4.1. This antibody is directed against the mouse H-2K$^k$ antigen (equivalent to human HLA) and does not cross react with any human tissues (Oi *et al.*, 1979). This was used as a negative control.

*Immunoperoxidase reaction*

The cryostat sections were incubated with one of the monoclonal anti-placental alkaline phosphatase antibodies (as neat tissue culture supernatant ($10\,\mu g\,ml^{-1}$) followed by peroxidase conjugated rabbit anti-mouse immunoglobulin (Dako Immunoglobulins a/s). The peroxidase reaction was developed using diaminobenzidine and hydrogen peroxide. Sections were then counterstained with haematoxylin and mounted for microscopical examination.

### Results

*Normal tissues*

Both monoclonal anti-placental alkaline phosphatase antibodies gave strong immunohistochemical reactions with human placental syncytiotrophoblast (Figure 1). A range of normal human tissues was tested using the same immunoperoxidase techniques (Table I) but neither antibody gave any reactivity. These normal tissues included biopsies of human testes which were clearly unreactive (see Figure 4) and thus contrasted with the reactivity of testicular tumours.

**Table I** Immunohistochemical staining of human tissues with monoclonal anti-placental alkaline phosphatase antibodies

| *Normal tissues* | *Number of samples* | *H17E2* | *D20L* |
|---|---|---|---|
| Breast | 4 | Negative | Negative |
| Brain | 1 | Negative | Negative |
| Skin | 4 | Negative | Negative |
| Kidney | 4 | Negative | Negative |
| Tonsil | 3 | Negative | Negative |
| Stomach | 1 | Negative | Negative |
| Colon | 5 | Negative | Negative |
| Liver | 1 | Negative | Negative |
| Oesophagus | 1 | Negative | Negative |
| Cervix | 2 | Negative | Negative |
| Lung | 4 | Negative | Negative |
| Testis | 4 | Negative | Negative |
| Ovary | 2 | Negative | Negative |
| Uterus | 1 | Negative | Negative |
| Placenta | 5 | Positive | Positive |

*Malignant tumours*

A wide range of biopsies from malignant tumours (Table II) was examined with both antibodies. Sixteen testicular tumours including seminomas and malignant teratomas (detailed in Table II) all gave positive reactions with H17E2 (Figures 2 and 3). D20L showed a more restricted staining pattern labelling only a small minority of cells in three cases of seminoma.

All the other tumours examined (Table II) were negative with both antibodies apart from one case of papillary serous adenocarcinoma of the ovary, a secondary poorly differentiated carcinoma of the ovary and a poorly differentiated carcinoma of the uterus (all strongly positive with H17E2) and three cases of colonic carcinoma (weakly and patchily stained with both antibodies).

Negative control antibody 11-4.1 produced a negative reaction against all tested tissues.

### Discussion

This study has identified a monoclonal antibody H17E2 that reacts strongly with germ cell tumours of the testis and some carcinomas of the female genital tract. No normal tissues showed any reactivity apart from placental syncytiotrophoblast against which the antibody was raised.

Previous studies using polyclonal antisera have provided evidence for the presence of placental alkaline phosphatase in a wide range of human neoplasms, e.g. seminoma of testis and tumours of breast, ovary, lung, stomach and pancreas (Fishman *et al.*, 1968; Stolbach *et al.*, 1979; Nathanson & Fishman, 1971; Uchida *et al.*, 1981; Wada *et al.*, 1979) as well as in some normal tissues such as cervix, lung and testes (Goldstein *et al.*, 1982; Chang *et al.*, 1980). It is known that placental alkaline phosphatase is a highly complex enzyme (Harris, 1982) which has been demonstrated in this study by the different patterns of staining of the two separate monoclonal antibodies directed against placental alkaline phosphatase. This study indicates that there is a particular epitope carried by the enzyme which is present in high concentration on normal human placental syncytiotrophoblast, testicular germ cell tumours and some tumours of the female genital tract. In addition it would appear that the antigenic forms recognised by both antibodies are present focally and at much lower concentrations in some

**Figure 1** Immunoperoxidase staining of term placenta using monoclonal antibody H17E2. Note strongly positive reaction with the membrane of syncytiotrophoblast.

**Figure 2** Immunoperoxidase staining of seminoma of testis with monoclonal antibody H17E2. Note strongly positive reaction.

**Figure 3** Immunoperoxidase staining of malignant teratoma (undifferentiated) of testis with monoclonal antibody H17E2. Note strongly positive reaction.

**Figure 4** Negative Immunoperoxidase staining of normal testis with antibody H17E2.

**Table II** Immunohistological reactions of testicular tumours and other neoplastic tissues with monoclonal anti-placental alkaline phosphatase antibodies

| *Testicular tumours* | *No. of cases* | *H17E2* | *D20L* |
|---|---|---|---|
| Seminoma **(Figure 2)** | 7 | Strongly positive | 4 cases negative 3 cases small minority of cells positive |
| Malignant teratoma: | | | |
| (a) Trophoblastic | 3 | Strongly positive | Negative |
| (b) Intermediate | 2 | Strongly positive | Negative |
| (c) Undifferentiated **(Figure 3)** | 2 | Strongly positive | Negative |
| Mixed tumours: | | | |
| (a) Malignant teratoma undifferentiated and seminoma | 1 | Strongly positive | Negative |
| (b) Malignant teratoma intermediate and seminoma | 1 | Strongly positive | Negative |
| Carcinomas: | | | |
| Squamous (skin) | 3 | Negative | Negative |
| Squamous (lung) | 3 | Negative | Negative |
| Basal cell (skin) | 4 | Negative | Negative |
| Malignant melanoma | 1 | Negative | Negative |
| Breast (ductal) | 6 | Negative | Negative |
| Kidney | 3 | Negative | Negative |
| Bladder | 1 | Negative | Negative |
| Colon | 5 | 3 cases weak and patchy positivity 2 cases negative | as H17E2 |
| Prostrate | 1 | Negative | Negative |
| Thyroid | 2 | Negative | Negative |
| Carcinoid | 3 | Negative | Negative |
| Ovary | 3 | 2 cases positive | Negative |
| Uterus | 1 | Strongly positive | Patchy positivity |
| Other neoplasms: | | | |
| Neuroblastoma | 1 | Negative | Negative |
| Ewing's sarcoma | 2 | Negative | Negative |
| Wilm's tumour | 2 | Negative | Negative |
| Leiomyosarcoma | 1 | Negative | Negative |

carcinomas of the colon. This is not surprising since one of the antibodies (D20L) was raised against a colonic carcinoma cell line. Two out of 6 colon carcinoma cell lines tested were positive with both antibodies while of 5 breast carcinoma lines 1 was positive (Travers & Bodmer, in preparation). It is interesting that antibody D20L showed a narrower distribution reacting only with some seminomas. It seems likely therefore, that the alkaline phosphatase found in germ cell tumours is the testis specific form of the enzyme rather than the placental form. This enzyme is expressed at low levels in the normal testis (Harris, 1982) presumably below the threshold of detectability by this study, but appears in these tumours at elevated levels. In the colon and uterine tumours, since D20L also shows reactivity, it is likely that there is a true ectopic expression of the placental isozyme.

For clinical use antibody H17E2 might be a useful adjunct in the histopathological diagnosis of testicular germ cell tumours providing that the patchy weak reactivity with several of the cases of colonic carcinoma is borne in mind. It could also be incorporated in a radioimmunoassay (RIA) or an enzyme linked immunoabsorbent assay (ELISA) for monitoring the status of patients with germ cell tumours as previously described by others using both polyclonal antisera and monoclonal antibodies (Lange, 1982; Millan *et al.*, 1982; McLaughlin *et al.*, 1982).

However, the potential importance of H17E2 is that in view of its specificity for placental alkaline phosphatase and the apparent absence of reactivity with normal tissues other than placenta, it could be used *in vivo* for tumour localisation studies. Previous studies from this and other centres

(Epenetos *et al.*, 1982; Farrands *et al.*, 1982; Berche *et al.*, 1982) using other tumour-associated monoclonal antibodies have shown promising results with this approach as a means of localising accurately the site and extent of tumours in individual patients. Furthermore, its apparent lack of reactivity with normal tissues renders it potentially useful as a therapeutic agent in the treatment of testicular germ cell tumours.

We thank J. Masters from the Institute of Urology, London, C. Furse from the Imperial Cancer Research Fund, London and J. Arklie from the MRC Molecular Biology Laboratory, Cambridge, without whose help this study would not have been possible. The immunohistological screening performed in Oxford for this study was generously supported by the Leukaemia Research Fund and the Wellcome Trust. K.G. holds the Gillson Scholarship in Pathology of the Society of Apothecaries of London.

### References

BERCHE, C., MACH, J.P., LUMBROSO, J.D. & 7 others. (1982). Tomoscintigraphy for detecting gastrointestinal and medullary thyroid cancers: first clinical results using radiolabelled monoclonal antibodies against carcinoembryonic antigen. *Br. Med. J.*, **285**, 1447.

BUDGER, K.S. & SUSSMAN, H.H. (1976). Structural evidence that human liver and placental alkaline phosphatase isoenzymes are coded by different genes. *Proc. Natl Acad. Sci.*, **73**, 2201.

CHANG, C.H., ANGELLIS, D. & FISHMAN, W.H. (1980). Presence of the rare D variant heat-stable placental type alkaline phosphatase in normal human testis. *Cancer Res.*, **40**, 1506.

EPENETOS, A.A., BRITTON, K.E., MATHER, S. & 8 others. (1982). Targeting of $^{123}$I labelled tumour associated monoclonal antibodies to tumours of patients with ovarian, breast and gastrointestinal malignancies. *Lancet*, **ii**, 999.

FARRANDS, P.A., PERKINS, A.C., PIMM, M.V. & 4 others. (1982). Radioimmunodetection of human colorectal cancers by an anti-tumour monoclonal antibody. *Lancet*, **ii**, 397.

FISHMAN, W.H. INGLIS, N.R., GREEN, S. & 6 others. (1968). Immunology and biochemistry of Regan isoenzyme of alkaline phosphatase in human cancer. *Nature*, **219**, 696.

FISHMAN, L., MIGAYAMA, H., DRISCOLL, S.G. & FISHMAN, W.H. (1976). Developmental phase-specific alkaline phosphatase isoenzymes of human placenta and their occurrence in human cancer. *Cancer Res.*, **36**, 2268.

GOLDSTEIN, D.J., ROGERS, C. & HARRIS, H. (1982). Evolution of alkaline phosphatases in primates. *Proc. Natl Acad. Sci.*, **79**, 879.

HARRIS, H. (1982). Multilocus enzyme systems and the evolution of gene expression. The alkaline phosphatases as a mouse model example. *The Harvey Lectures*, Series 76, p. 75.

KOHLER, G. & MILSTEIN, C. (1975). Continuous cultures of fused cells secreting antibody of predefined specificity. *Nature*, **256**, 495.

LANGE, P.H., MILLAN, J.L., STIGBRAND, T., VESSELLA, R.L., RUOSLAHTI, E. & FISHMAN, W.H. (1982). Placental alkaline phosphatase as a tumour marker for seminoma. *Cancer Res.*, **42**, 3244.

McLAUGHLIN, P.J., CHENG, M.H. & JOHNSON, P.M. (1982). Expression on cultured human tumour cells of placental trophoblast membrane antigens and placental alkaline phosphatase defined by monoclonal antibodies. *Int. J. Cancer*, **30**, 21.

MILLAN, J.L., STIGBRAND, T., RUOSLAHTI, E. & FISHMAN, W.H. (1982). Characterisation and use of an allotype-specific monoclonal antibody to placental alkaline phosphatase in the study of cancer related phosphatase polymorphism. *Cancer Res.*, **42**, 2444.

NATHANSON, L. & FISHMAN, W.H. (1971). New observations on the Regan isoenzyme of alkaline phosphatase in cancer patients. *Cancer*, **27**, 1388.

OI, V.T., JONES, P.P., GODING, J.W. & HERZENBERG, L.A. (1979). Current properties of monoclonal antibodies to base Ig Allotype, $H_2$ and Ia antigens. *Curr. Topic Microbiol. Immunol.*, **81**, 115.

STOLBACH, L.L., KRANT, M.J. & FISHMAN, W.J. (1969). Ectopic production of an alkaline phosphatase isoenzyme in patients with cancer. *N. Engl J. Med.*, **281**, 757.

STRAGAARD, J.J., BERGERAT, J.P., WHITE, A.R., HOKANSON, J. & DREWINO, B. (1980). Biological and cell kinetic properties of a human colonic adenocarcinoma (LoVo) grown in athymic mice. *Cancer Res.*, **40**, 2846.

UCHIDA, T., SHIMODA, T., MIYATA, H. & 6 others. (1981). Immunoperoxidase study of alkaline phosphatase in testicular tumour. *Cancer*, **48**, 1455.

WADA, H.G., SHINDELMAN, J.E., ORTMEYER, A.E. & SUSSMAN, H.H. (1979). Demonstration of placental alkaline phosphatase in human breast cancer. *Int. J. Cancer*, **23**, 781.